# Footprints: Stamping hallmarks of lung cancer with patient-derived models, from molecular mechanisms to clinical translation

**DOI:** 10.3389/fbioe.2023.1132940

**Published:** 2023-02-21

**Authors:** Yang Song, Yadong Wang, Ai Guan, Jianchao Xue, Bowen Li, Zhicheng Huang, Zhibo Zheng, Naixin Liang, Yanlian Yang, Shanqing Li

**Affiliations:** ^1^ Department of Thoracic Surgery, Peking Union Medical College Hospital, Chinese Academy of Medical Sciences, Beijing, China; ^2^ Peking Union Medical College Hospital, Chinese Academy of Medical Sciences, Beijing, China; ^3^ Department of International Medical Services, Peking Union Medical College Hospital, Chinese Academy of Medical Sciences, Beijing, China; ^4^ CAS Key Laboratory of Biological Effects of Nanomaterials and Nanosafety, CAS Key Laboratory of Standardization and Measurement for Nanotechnology, CAS Center for Excellence in Nanoscience, National Center for Nanoscience and Technology, Beijing, China

**Keywords:** Hallmarks of cancer, NSCLC, SCLC, clinical application, precision medicine

## Abstract

The conventional two-dimensional (2D) tumor cell lines in Petri dishes have played an important role in revealing the molecular biological mechanism of lung cancer. However, they cannot adequately recapitulate the complex biological systems and clinical outcomes of lung cancer. The three-dimensional (3D) cell culture enables the possible 3D cell interactions and the complex 3D systems with co-culture of different cells mimicking the tumor microenvironments (TME). In this regard, patient-derived models, mainly patient-derived tumor xenograft (PDX) and patient-derived organoids discussed hereby, are with higher biological fidelity of lung cancer, and regarded as more faithful preclinical models. The significant *Hallmarks of Cancer* is believed to be the most comprehensive coverage of current research on tumor biological characteristics. Therefore, this review aims to present and discuss the application of different patient-derived lung cancer models from molecular mechanisms to clinical translation with regards to the dimensions of different hallmarks, and to look to the prospects of these patient-derived lung cancer models.

## 1 Introduction

Lung cancer is one of the most common malignancy burdens, with an estimated 1.8 million deaths worldwide in 2020. Globally, 21.5% of cancer-related deaths in men and 13.7% in women are attributable to lung cancer (Sung et al., 2021). The conventional treatments, including radiotherapy, chemotherapy and anti-angiogenic therapy, showed limited efficacy in improving the outcomes of patients with lung cancer (Goldstraw et al., 2016). Fortunately, the identification of oncogenic driver mutations and the development of corresponding targeted agents as well as remarkable breakthroughs in cancer immunotherapy have dramatically changed the treatment landscape of lung cancer (Wang et al., 2021). Despite this, many important issues such as mechanisms of tumorigenesis, development of biomarkers, drug screening, and individualized precision medicine remain to be addressed. Patient-derived lung cancer models play an increasingly important role in elucidating the above issues (Hynds et al., 2021).

The conventional two-dimensional (2D) cell culture model is very convenient and significant in revealing the molecular biological mechanism. However, the lack of information in 2D cell culture model in tumor genotype and phenotype heterogeneity, three-dimensional (3D) cell-cell interactions, and even the cell-matrix interactions render this model poor predictive value for the clinical efficacy. With the progress of 3D cell cuture technologies, the tumor microenvironment (TME) could be mimicked and retained to the maximum extent. Patient-derived models are drawing more and more attention and showing great potential in translational clinical cancer research.

Patient-derived lung cancer models are built from the precious specimens of individual patients. While faithfully retaining the genetic characteristics of the parental tumor, the disease heterogeneity between individual patients. Therefore, it helps to comprehensively explore the occurrence, development, and clinical outcomes of lung malignancies (Huo et al., 2020). The main patient-derived lung cancer models include patient-derived organoids (PDO), patient-derived tumor xenograft model (PDX), circulating tumor cell (CTC)-derived model, etc.

PDX originated in the 1970s (Schroeder), and the definition has been continuously expanded. It currently refers to a preclinical model established by transplanting fresh tissue from patients (including but not limited to biopsy or surgically resected tumor specimens, and enriched CTCs) into immunodeficient mice. Different from the *in vitro* models, PDX is believed to retain the tumor microenvironment and epigenetic characteristics, which are closely related to tumorigenesis, tumor invasion and metastasis, and the efficacy of the antitumor therapies. Hence there is a higher success rate of clinical translation.

PDO is a 3D structure culture formed from enriched patient cancer cells. It also has the characteristics of genetic stability, as well as self-renewal and drug sensitivity, and can highly simulate human organs in structure and function. Its key feature is also the faith preservation of genomic changes of the parental tumor, but with shorter modeling time and allowing for gene editing to some extent.

**FIGURE 1:**
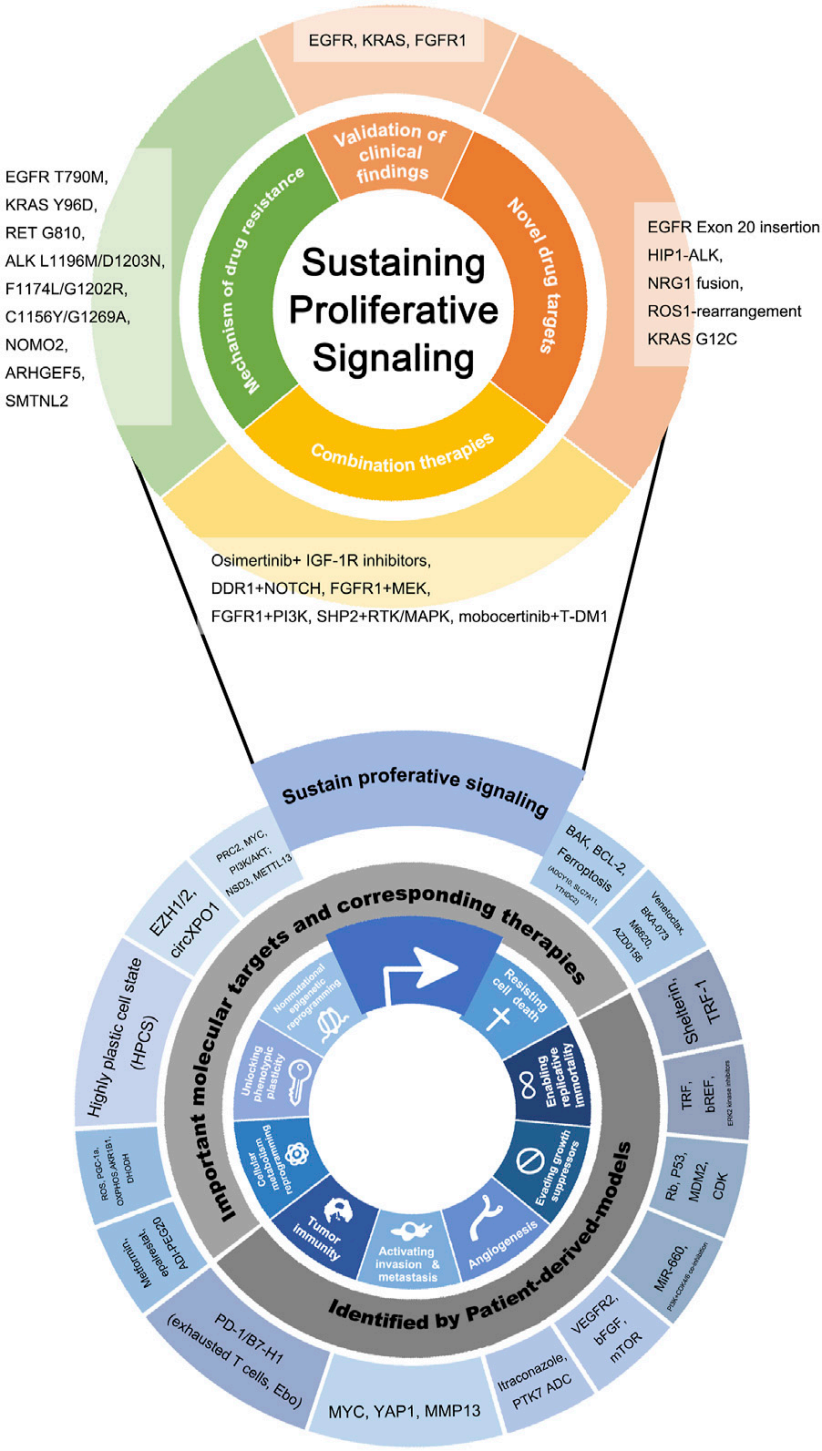
Novel molecular targets and novel drugs discovered or validated by patient-derived lung cancer models, listed according to hallmarks of cancer as the framework. At the bottom of the figure, the inward ring represents the framework of this review and is composed of the hallmarks of cancer put forward by Hanahan et al. The hallmarks that are the most closely relevant to patient-derived models are selected. The outward ring represents significant genes, molecules, drugs, and biological behaviors involved in patient-derived models related basic and clinical research. Findings on Sustaining proliferative signaling were particularly plentiful and clinically meaningful, so the partial “zoom-in” was performed and the findings were subdivided by clinical application.

**FIGURE 2:**
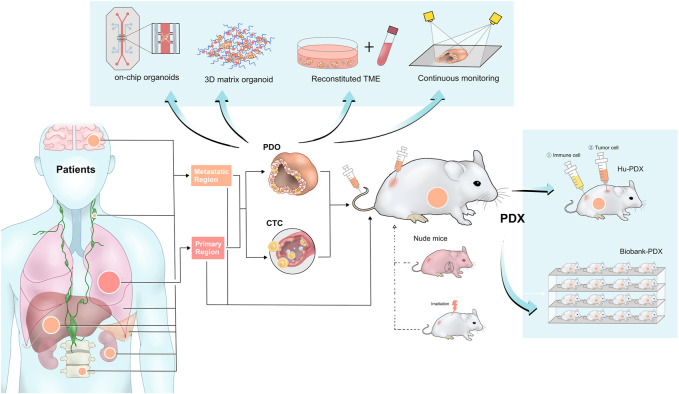
Different patient-derived lung cancer models and their cutting-edge technology foresight. PDX models can be established directly from primary tumors, metastatic lesions, pleural fluid, and cerebrospinal fluid. Meanwhile, patient-derived lung cancer specimens can be used to isolate CTC or culture organoid. PDX models can subsequently be established from treated organoid or CTC, indicating the different patient-derived lung cancer models are complementary and capable of mutual transformation. The blue-shaded boxes represent the Frontier area of research in patient-derived lung cancer models, including organoid culture technology, exploration of the possible mechanisms of immunotherapy in the Hu-PDX model, and highly promising biobank. PDX, patient-derived tumor xenograft; PDO, patient-derived organoid; CTC, circulating tumor cells; Hu-PDX, humanized PDX; TME, tumor microenvironment.

With the deepening of cancer research, *the Hallmarks of Cancer* put forward by Hanahan et al. summarize the enormous complexity of cancer phenotypes and genotypes into a series of basic principles, which provide great inspiration for original research works (Hanahan and Weinberg, 2011; Hanahan, 2022). As the hallmarks are comprehensively structural descriptions of molecular mechanisms and corresponding clinical manifestations of cancer, taking these hallmarks as clues, this review demonstrates and discusses the important research findings relating to the hallmarks of lung cancer (Figure 1; Table 1), while the research gaps still existing on this "treasure map" is also suggested. After a comprehensive review of the past, in the second part, we focus on the combination of patient-derived lung cancer models with new technologies, and the upgrade and expansion of the technology are demonstrated (Figure 2). Emerging important breakthroughs and more patient benefits are expected in the future.

**TABLE 1:** Recent key findings on the hallmarks of lung cancer, obtained from or validated by patient-derived models.

Hallmarks	Author, year	Cancer type	Patient-derived model	Novel targets/drug identified	Main conclusion	Clinical phases
Sustaining proliferative signaling	Fang, 2014	NSCLC	PDX	HIP1-ALK	A novel ALK rearrangement mutation, HIP1-ALK fusion, was found to be sensitive to crizotinib	clinical use
	Wang, 2019	NSCLC	PDO, PDX	HER2 exon 20 insertion/pyrotinib	Pyrotinib showed activity against NSCLC with HER2 exon 20 mutations in both PDO and PDX models, providing preliminary data from further clinical trials	preclinical stage
	Schueler, 2019	NSCLC	PDX	NOMO2, ARHGEF5, SMTNL2	NOMO2, ARHGEF5 and SMTNL2 were associated with EGFR TKI aquired resistance	basic research
	Yun, 2020	NSCLC	PDX	ROS1/TRK/ALK, repotrectinib	ROS1/TRK/ALK-TKI repotrectinib showed effects on both treatment-naïve and solvent-front-mutant ROS1-rearranged NSCLC, and it had blood-brain barrier penetrating properties	clinical use
	Yun, 2020	NSCLC	patient-derived cell line, PDO, PDX	EGFR Exon 20 insertion/amivantamab (JNJ-61186372)	Amivantamab (JNJ-61186372), an EGFR-MET bispecific antibody, was effective against EGFR Exon 20 insertion mutation	clinical use
	Solomon, 2020	NSCLC	CDC, PDX	RET G810	The first definitive RET inhibitor resistance mutation, RET G810, was identified	clinical use
	Gonzalvez, 2021	NSCLC	patient-derived cell line, LCO, PDX	EGFR Exon 20 insertion/mobocertinib (TAK-788)	Mobocertinib (TAK-788) was effective against EGFR Exon 20 insertion mutation	clinical use
	Tanaka, 2021	NSCLC	PDX	KRAS Y96D	KRAS G12C associated resistant mutations converged to reactivated RAS-MAPK signaling. A new KRAS Switch-II Pocket Mutation KRAS Y96D contributing to this resistance was identified	basic research
	Wang, 2019	SCLC	PDX	RTK, pERK	Abnormal RTK-related genes and pERK expression were associated with SCLC chemotherapy resistance	basic research
	Augert, 2019	SCLC	PDX	LSD1	LSD1 inhibitor achieveD tumor regression and restore neuroendocrine phenotype in SCLC through reactivating NOTCH.	basic research
	Giffin, 2021	SCLC	PDX	DLL3/NOTCH, AMG757	AMG757 demonstrated efficacy aginst SCLC by simultaneously targeting DLL3 and engaging T cells	basic research
	Huo, 2020	Lung squamous cell carcinoma	patient-derived cell line, PDO, PDX	FGFR1+MEK, FGFR1+PI3K	for lung squamous cell carcinoma resistant to FGFR1 inhibitors, FGFR1 inhibitor BGJ398 was combined with MEK inhibitor (trametinib) or PI3K inhibitor (BKM120) to overcome the single-drug resistance	basic research
	Wang, 2020	Lung cancer	PDX	IGF-1R inhibition + osimertinib	In AXL-low expressing EGFR-mutated lung cancer, osimertinib resistance was driven by IGF-1R activation. The combination of IGF-1R inhibitors and osimertinib reversed the resistance	basic research
	Li, 2020	Lung cancer	PDX	HER2/T-DM1	In lung cancers with amplifying or mutated ERBB2, T-DM1 was effective	preclinical stage
	Recondo, 2020	Lung cancer	patient-derived cell line	ALK	Three mutations in the ALK kinase domain mediated to lorlatinib resistance, ALK L1196M/D1203N, F1174L/G1202R, and C1156Y/G1269A, were identified	clinical use
	Zhao, 2021	lung cancer	PDX	KRAS G12C	Acquired oncogenic KRAS, NRAS or BRAF mutations were associated with KRAS G12C inhibitor resistance	basic research
	Liu, 2021	Lung cancer	PDX	SHP2+RTK/MAPK	SHP2 inhibitor TNO155 was synergistic with RTK/MAPK inhibitors	basic research
	Odintsov, 2021	Lung cancer	PDX	NRG1 fusion mutation/rapamycin	Tumorigenesis of NRG1 fusion mutation was achieved by HER3 and MTOR signaling. Rapamycin showed inhibitory effects of this possible mechanism	basic research
	Ambrogio, 2016	Lung adenocarcinoma	PDX	DDR1+NOTCH	Dual inhibition of DDR1 and Notch signaling was found to induce lung adenocarcinoma tumor regression	basic research
	Makimoto, 2019	Lung adenocarcinoma	patient-derived cell line + PDX	ALK	Possible mechanism of ALK TKI resistance is the evolution of high TMB and tumor heterogeneity	basic research
	Hallin, 2020	Lung adenocarcinoma	PDX	KRAS G12C/MRTX849	MRTX849 was the world’s second KRAS G12C-targeted drug	clinical use
	Han, 2021	Lung adenocarcinoma	PDX	HER2 exon 20 insertion/mobocertinib T-DM1	Combination of mobocertinib (TAK-788) and T-DM1 was proved to have a strong antitumor effect on HER2 exon 20 insertion-mutant lung cancer	clinical use
Resisting cell death	Baschnagel, 2021	NSCLC with brain metastasis	PDX	DNA DSB repair/M6620 + radiotherapy	M6620 inhibited the DNA double strand break repairs. The combination of M6620 and radiotherapy showed synergistic effects	basic research
	Riches, 2020	SCLC	CTC	ferroptosis	Non-NE SCLC was ferroptosis-sensitive	basic research
	Bebber, 2021	SCLC	PDX	BCL-2/venetoclax	SCLC subset with high BCL-2 expression was sensitive to venetoclax	basic research
	Park, 2021	Pan-cancer	PDX	ATM/AZD0156 + radiotherapy	SHP2 inhibitor TNO155 for treatment with RTK/MAPK inhibitors	basic research
	Lochmann, 2018	Lung cancer	PDX	Bak/BKA-073 + venetoclax	Bak initiated apoptosis by activating the BH3 death domain. Bak activator BKA-073 was synergistic with venetoclax against lung cancer	basic research
	Ye, 2020	Lung adenorcarcinoma	PDX	ferroptosis, YTHDC2	Some lung adenocarcinoma was sensitive to ferroptosis inducers because SLC7A11 was a direct target of YTHDC2, which inhibited cystine uptake	basic research
	Ma, 2021	Lung adenocarcinoma	human cell-derived xenograft, PDX	ferroptosis, ADCY10	Advanced lung adenocarcinomas with high ADCY10 expression were sensitive to ferroptosis	basic research
	Zhang, 2021	Lung adenocarcinoma	PDX	ferroptosis-inducing agent + radiotherapy	Ferroptosis was associated with the radiotherapy-induced cancer cell death, and ferroptosis-inducing agents and radiation had a synergistic effect	basic research
Epigenetic reprogramming	Gardner, 2017	SCLC	PDX	EZH2	Chemotherapy resistance was associated with an EZH2-mediated histone modification H3K27me3, and EZH2 inhibitors prevent SCLC acquiring resistance to standard chemotherapy	basic research
	Liu, 2019	Lung cancer	PDX	METT13	Double methylation catalysis of eEF1A by METTL13 promoted RAS-driven tumorigenesis	basic research
	Huang, 2020	Lung adenocarcinoma	PDX	circXPO1	High circXPO1 expression was associated with poor overall survival, and targeting circXPO1 inhibited tumor growth	basic research
	Quintanal-Villalonga, 2021	Lung adenocarcinoma	PDX	PI3K/AKT inhibitors + osimertinib against the NE transformation	NE transformation and the squamous cell carcinoma transformation are mainly mediated by transcriptional reprogramming. Up-regulation of PRC2, MYC, and PI3K/AKT pathway gene expression was observed in both transdifferentiation forms	basic research
	Quintanal-Villalonga, 2021	Lung adenocarcinoma	PDX	EZH1/2 inhibitor + osimertinib resensitized the resistant squamous-like tumor		basic research
	Yuan, 2021	Lung squamous cell carcinoma	PDX	NSD3 (T1232A)	The variant NSD3 (T1232A) caused the nearby oncogenes to be turned on by dimethylation of histone H3 lysine 36 (H3K36), which drives the progression of lung squamous cell. carcinoma	basic research
Cellular metabolism reprogramming	Cruz-Bermudez, 2019	NSCLC	PDX	PGC-1α/OXPHOS, metformin	ROS-mediated increase in PGC-1a was associated with cisplatin resistance in NSCLC. PGC-1α interference or OXPHOS inhibition using metformin delayed or counteracted cisplatin resistance	basic research
	Li, 2019	SCLC	PDX	DHODH	DHODH is the catalytic enzyme in the *de novo* pyrimidine synthesis pathway. Inhibition of DHODH can inhibit the growth of SCLC.	basic research
	Chalishazar, 2019	SCLC	Human cell line xenografts, PDX	ADI-PEG20	For MYC-driven SCLC, adding ADI-PEG 20 to intervene arginine synthesis exhibited antitumor properties beyond conventional chemotherapy	basic research
	Zhang, 2021	Lung cancer	PDX	AKR1B1/epalrestat	AKR1B1 promoted glutathione *de novo* synthesis by activating STAT3, leading to ROS scavenging and ultimately to EGFR-TKI drug resistance. AKR1B1 inhibitor epalrestat delayed the EGFR-TKI resistance	basic research
Evading growth suppressors	Shi, 2018	Lung squamous cell carcinoma	PDX	PI3K + CDK4/6	In a model with a PIK3CA mutation, combined inhibition of PI3K and CDK4/6 resulted in greater antitumor effects than either monotherapy	basic research
	Moro, 2019	Lung cancer	PDX	MDM2-P53	Coated cationic lipid-nanoparticles entrapping miR-660 reduced cancer cell proliferation by inhibiting MDM2 and restoring p53 function and its downstream effectors such as p21	basic research
Angiogenesis	Zhang, 2017	NSCLC	PDX	Itraconazole (ITA)	ITA was validated to be a multi-target antiangiogenic agent	basic research
	Damelin, 2017	NSCLC	PDX	PTK7	PTK7 ADC specifically reduced the frequency of tumor-initiating cells (TICs), and exhibited inhibition of angiogenesis	basic research
Cell immortality	Bejarano, 2019	Lung cancer	PDX	TRF-1	Telomerase shelterin is TRF-1-dependent, TRF-1 inhibitors, or inhibitors of bREF and ERK2 kinase, induced telomere damage and inhibit tumor growth	basic research
Invasion and metastasis	Shih, 2020	Lung adenocarcinoma	PDX	MYC, YAP1, MMP13	Overexpression of MYC, YAP1 or MMP13 increased the incidence of brain metastase	basic research
Tumor immunity	Sanmamed, 2021	NSCLC	Hu-PDX	PD1/B7H1	A distinct burnt-out CD8^+^ TIL subset (Ebo) was found in TME, and it was associated with the immune checkpoint inhibitors resistance	clinical use
Phenotypic plasticity	Marjanovic, 2020	Lung adenocarcinoma	PDX, 3D tumor spheres	HPCS cluster	Highly plastic cell state (HPCS) subpopulations had characteristics of tissue stem cells and CSCs. HPCS was related to the NE conversion of lung adenocarcinoma	basic research

## 2 Embodiment of the hallmarks of cancer from patient-derived models

### 2.1 Sustaining proliferative signaling

Sustaining proliferative signaling is the first proposed hallmark of cancer, which refers to the feature that cancer cells acquire sustained proliferation signals and become masters of their own destiny. The binding of growth factors leads to the continuous activation of intracellular tyrosine kinase domains as the common pathway (Hanahan and Weinberg, 2011). In lung cancer, based on these important targets and signaling pathways, EGFR, KRAS, ALK, ROS1, RET, BRAF, MET, HER-2, and NTRK inhibitors have been applied, rewriting the era of the treatment of lung cancer. Since 2015, there have been many PDX models of lung cancer tested, and their therapeutic response to EGFR actionable mutation inhibitors is consistent with that observed in corresponding patients. This is also an important way to validate the fidelity of PDX models (Stewart et al., 2015).

#### 2.1.1 Decipher the molecular mechanism of targeted therapy resistance

The development of drug resistance seems to be the inevitable result of targeted therapies, and it is indeed the biggest clinical challenge. Drug resistance has been demonstrated and verified in the PDX model. The lung adenocarcinoma PDX model with EGFR exon 19 deletion and EGFR T790M mutation was resistant to gefitinib/erlotinib, but responded to cetuximab (Martin et al., 2016). Whereas, the non-small cell lung cancer (NSCLC) PDX model with KRAS and FGFR1 amplification was resistant to gefitinib (Zhang et al., 2013).

To conquer the resistance to targeted therapy, patient-derived models helped to decipher the mechanism of drug resistance. Through sequencing on longitudinal tumor biopsies from lung cancer patients resistant to third-generation ALK inhibitor lorlatinib, combined with the *in vivo* studies on patient-derived cell lines, Recondo et al. not only found that epithelial-mesenchymal transition (EMT) might mediate lorlatinib resistance, but also identified three mutations in the ALK kinase domain featuring the drug resistance: ALK L1196M/D1203N, F1174L/G1202R, and C1156Y/G1269A (Recondo et al., 2020). Schueler et al. induced drug resistance in an EGFR-driven PDX model of NSCLC by continuous treatment with gefitinib *in vivo*. Three genes associated with acquired resistance to EGFR-tyrosine kinase inhibitor (TKI) were identified by whole-exome sequencing and reverse-phase protein array (RPPA): NOMO2, ARHGEF5, and SMTNL2 (Schueler et al., 2019). From an advanced ALK-positive and alectinib-resistant lung adenocarcinoma patient, following the clinical process of metastasis and drug resistance, researchers developed patient-derived cell lines and PDX from the primary tumor, pleural effusion, and distant metastases, and studied their molecular biological characteristics respectively. They found that the possible mechanism of ALK TKI resistance is the evolution of high tumor mutation burden (TMB) and tumor heterogeneity (Makimoto et al., 2019). After this, in another PDX model study involving 43 patients with KRAS G12C inhibitor resistance, it was identified that subclonal events emerging during KRAS G12C inhibitor resistance were acquired oncogenic KRAS, NRAS, or BRAF mutations (Zhao et al., 2021a). On this basis, it is identified the aforementioned KRAS G12C resistant mutations all converged to reactivated RAS-MAPK signaling, and proposed a new KRAS Switch-II Pocket Mutation KRAS Y96D contributing to this resistance (Tanaka et al., 2021). This series of studies is an outstanding example that the patient-derived model is an indispensable baton in the research on deciphering the mechanism of targeted therapy resistance, and continuously participates in the process of identifying a specific molecular target. In addition, the first definitive RET inhibitor resistance mutation, RET G810, was also identified by screening CTCs enriched from corresponding resistant patients, followed by validation in a PDX model (Solomon et al., 2020).

#### 2.1.2 Finding new potential drug targets and combination therapies

To address the challenge of targeted therapy resistance, researchers are committed to finding potential new drugs or new drug targets, or to proposing important combination therapy options. PDX was used for preclinical testing of the world’s second KRAS G12C-targeted drug, MRTX849 (Hallin et al., 2020). For the mutation EGFR Exon 20 insertion, the novel EGFR TKI mobocertinib (TAK-788), as well as Amivantamab (JNJ-61186372), an EGFR-MET bispecific antibody, showed antitumor activity in the patient-derived cell line, PDO, and PDX (Yun et al., 2020a). Through the screening of numerous NSCLC PDX models, a novel anaplastic lymphoma kinase (ALK) rearrangement mutation, huntingtin interacting protein 1 (HIP1)-ALK fusion gene, was found to be sensitive to crizotinib (Fang et al., 2014). In a series of studies, the researchers firstly found that the tumorigenesis of NRG1 fusion mutation was achieved by HER3 and mTOR signaling in an *in vitro* cell line model, and then verified the inhibitory and tumor suppressive effects of rapamycin on this possible mechanism in a PDX *in vivo* model (Odintsov et al., 2021). ROS1/TRK/ALK-TKI repotrectinib, in the PDX model, showed tumor-suppressive effects on both treatment-naïve and solvent-front-mutant ROS1-rearranged NSCLC. Especially because of its blood-brain barrier penetrating properties, it had a prominent effect in lung cancer patients with brain metastasis (Yun et al., 2020b).

In terms of the novel combination therapies, Wang et al. found that in AXL-low expressing EGFR-mutated lung cancer, osimertinib resistance was driven by insulin-like growth factor-1 receptor (IGF-1R) expression and phosphorylation. The combination of IGF-1R inhibitors and osimertinib in an *in vivo* PDX model can reverse the resistance (Wang et al., 2020). In a lung adenocarcinoma KRAS-mutated PDX model, dual inhibition of DDR1 and NOTCH signaling was found to induce tumor regression (Ambrogio et al., 2016). In a study of lung squamous cell carcinoma resistant to FGFR1 inhibitors, it was found that the FGFR1 inhibitor BGJ398 was combined with MEK inhibitor (trametinib) or PI3K inhibitor (BKM120) to overcome the BGJ398 single-drug resistance, and the results were consistent in the human-derived cell line, PDO, and PDX models (Huo et al., 2020). In a pan-cancer study, PDX biobank was used as an *in vivo* validation model to demonstrate the potentiation of SHP2 inhibitor TNO155 for treatment with RTK/MAPK inhibitors (Liu et al., 2021). Antibody-drug conjugates (ADC) combine the advantages of high specific targeting ability and potent killing effect to achieve precise and efficient killing of cancer cells. In lung cancers with amplifying or mutated ERBB2, ado-trastuzumab emtansine (T-DM1) was also validated in the PDX model before becoming the first ADC approved in China (Li et al., 2020). On this basis, the combination of mobocertinib (TAK-788) mentioned above and T-DM1 was proved to have a strong antitumor effect on HER2 exon 20 insertion-mutant lung cancer (Han et al., 2021).

In terms of small cell lung cancer (SCLC) research, one of the main challenges is the extremely limited availability of tumor specimens. Wang et al. established PDX from valuable specimens obtained from bronchoscopy biopsies. In this way PDX was established in more than half of the patients and sufficient tumor tissue for downstream research was obtained. They found that abnormal RTK-related genes and pERK expression were associated with SCLC chemotherapy resistance (Wang et al., 2019). Augert et al. found that LSD1 inhibitor may achieve tumor regression and restore neuroendocrine (NE) phenotype in SCLC PDX by reactivating the NOTCH pathway (Augert et al., 2019). Giffin et al. found that AMG757 demonstrated preliminary efficacy and acceptable safety in SCLC preclinical studies by simultaneously targeting DLL3 and engaging T cells (Giffin et al., 2021).

### 2.2 Resisting cell death and enabling replicative immortality

#### 2.2.1 Resisting cell death

Cell death is an essential biological process playing an important role in embryonic development, maintenance of tissue homeostasis, and host immune defense. However, tumor cells show resistance to cell death when they experience various internal and external pressures such as physiological stress and anti-tumor therapy.

The BCL-2 inhibitor, venetoclax, was firstly demonstrated to have antitumor activity in hematological tumors, and its remarkable effect inspired researchers to explore its role in other cancers. High-throughput screening of venetoclax-sensitive SCLC subsets was applied using cell lines, and the antitumor was verified in the PDX model of SCLC with high BCL-2 expression (Lochmann et al., 2018). Another BCL-2 family member, Bak, initiates apoptosis by activating the BH3 death domain. Park et al. screened out the Bak activator BKA-073 from the UCSF DOCK 6.1 program suite and the National Cancer Institute (NCI) chemical library, and it has been shown to be synergistic with venetoclax in the PDX model of NSCLC and SCLC (Park et al., 2021).

In addition to the classic BCL-2 pathway, another cell death-related research hotspot in recent years is ferroptosis. Ferroptosis is a newly discovered regulated form of cell death in which the inhibition of the cystine-glutamate anti-transport system leads to impaired cystine uptake, thereby inducing ferroptosis. Zhang et al. used a human cell-derived xenograft and PDX model to verify that advanced lung adenocarcinomas with high ADCY10 expression were sensitive to ferroptosis (Zhang et al., 2021a). When studying the tumor suppression of m6A reader YTHDC2 in lung adenocarcinoma, Ma et al. also found that the PDX model of lung adenocarcinoma was sensitive to ferroptosis inducers because SLC7A11 (the catalytic subunit of system X_C_-) was a direct target of YTHDC2, which inhibited cystine uptake (Ma et al., 2021). Ye et al. found that ferroptosis was also partly responsible for the radiotherapy-induced cancer cell death, and that small-molecule ferroptosis-inducing agents and radiation have a synergistic effect in a PDX model. Therefore, ferroptosis inducers may be effective radiosensitizers, potentially expanding the efficacy and indications of radiotherapy (Ye et al., 2020). Bebber et al. found that non-neuroendocrine SCLC (non-NE SCLC) were ferroptosis-sensitive, but not the neuroendocrine (NE) SCLC (Bebber et al., 2021).

NSCLC tumors have significant defects in DNA damage response (DDR) genes, Baschnagel et al. used an NSCLC brain metastasis PDX model and found that M6620 inhibited the DNA double strand break (DSB) repairs. The combination of M6620 and radiotherapy showed synergistic effects (Baschnagel et al., 2021). Similarly, Riches et al. found that the ATM-selective small molecule inhibitor AZD0156 enhanced the inhibitory effect of radiotherapy on tumor growth by eliminating radiotherapy-induced activation of the ATM signaling pathway, in both the NSCLC cell line xenograft model and the PDX model (Riches et al., 2020).

#### 2.2.2 Enabling replicative immortality

Normal cells can only go through a limited growth-division cycle (due to senescence, cell crisis, and cell death), whereas in addition to resisting cell death, tumor cells simultaneously exhibit indefinite cancer growth through telomere maintenance. Most previous studies have focused on the inhibition of abnormally activated telomerase. However, recent researches suggested that targeting the telomere protective complex, or the shelterin directly might be a more effective strategy. Since the shelterin is TRF-1-dependent, TRF-1 inhibitors, or inhibitors of bREF and ERK2 kinase, which are associated with TRF-1 phosphorylation, might induce telomere damage and inhibit tumor growth. This hypothesis has been confirmed in the glioblastoma PDX model and holds promise in lung cancer (Bejarano et al., 2019).

### 2.3 Evading growth suppressors

Evading growth suppressors refers to the feature of tumor allowing continuous cell proliferation, as a result of lacking the gatekeepers that strictly regulate the cell growth cycle. The most typical and well-known genes about this hallmark are Rb and P53. Tumor-bearing mice could spontaneously form SCLC after knocking out p53 and Rb genes (Meuwissen et al., 2003), and whole-genome sequencing results showed that almost all clinical SCLC tissue samples were with deletion of P53 and Rb genes (George et al., 2015). In in vivo experiments with lung cancer PDX, it was found that coated cationic lipid-nanoparticles entrapping miR-660 reduced cancer cell proliferation by inhibiting MDM2 and restoring p53 function and its downstream effectors such as p21 (Moro et al., 2019).

Cyclin-dependent Kinase (CDK) inhibitors that regulate the growth cycle are another important treatment based on this hallmark. Alterations in PI3K can be detected in more than 50% of lung squamous cell carcinomas, but PI3K inhibitor monotherapy has limited efficacy in lung squamous cell carcinomas. In a model with a PIK3CA mutation, Shi found that combined inhibition of PI3K and CDK4/6 resulted in greater antitumor effects than either monotherapy, providing preclinical evidence for further clinical trials (Shi et al., 2018). This once again demonstrates that, in addition to discovering new drugs, *in vivo* PDX models can aid in the discovery of new combination therapy options, as previously described.

### 2.4 Inducing angiogenesis and activating invasion and metastasis

#### 2.4.1 Inducing angiogenesis

Current anti-angiogenic drugs mainly target vascular endothelial growth factor (VEGF) or receptor tyrosine kinases. Anti-angiogenesis agents have exhibited valuable clinical efficacy for lung cancer patients (Li et al., 2022). PDX models of human lung cancer have been widely used in validating the anti-angiogenic effects. For example, the antifungal drug Itraconazole (ITA) has been recently validated to be a multi-target antiangiogenic agent capable of affecting multiple angiogenic stimulating signal pathways, including VEGF, basic fibroblast growth factor (bFGF), glycosylation of vascular endothelial growth factor receptor 2 (VEGFR2) and mammalian target of rapamycin (mTOR) pathway. Zhang et al. constructed ITA’s polymer micelles (IP2K) and albumin nanoparticles (IBSA). They did not inhibit the growth of NSCLC tumors *in vitro*, but showed an antiangiogenic effect in the NSCLC PDX model (Zhang et al., 2017). Damelin et al. applied the PDX model to study PTK7 ADC and found that PTK7 ADC not only specifically reduced the frequency of tumor-initiating cells (TICs), but also exhibited other tumor suppression effects, including the inhibition of angiogenesis and the stimulation of immune cells (Damelin et al., 2017).

#### 2.4.2 Activating invasion and metastasis

SCLC CTCs spontaneously formed "tumor spheres" in culture, even without the need for lung stem cell culture medium. The phenomenon that CTCs grew in clusters rather than single cells might explain the commonly observed chemoresistance in patients with relapsed SCLC (Klameth et al., 2017).

Brain metastasis is an important metastatic pathway of lung adenocarcinoma, and is closely related to the poorer living conditions and the risk of death of patients. In order to identify the driver genes of this important metastasis pathway at the molecular level, the researchers first screened out mutation candidates with higher frequency in brain metastatic lung cancer by case-control analysis and then used the PDX model to perform *in vivo* experiments on the screened high-frequency mutations. They validated that overexpression of MYC, YAP1, or MMP13 increases the incidence of brain metastases (Shih et al., 2020).

### 2.5 Tumor-promoting inflammation and avoiding immune destruction

The study of tumor immunity is greatly limited due to the intrinsic limitations of the PDX, in which highly combined immunodeficient NOD-SCID or NSG mice were utilized. However, in recent years, the humanized PDX (Hu-PDX) with mice reconstituted with human immune systems has emerged, which makes it possible to partially reconstitute human immunity by implanting human hematopoietic cells, lymphocytes or tissues into immunodeficient mice system. For example, Meraz et al. combined CD34^+^ stem cells (CD34^+^ HSC) from fresh umbilical cord blood with lung cancer PDX, in which human T cells, B cells, NK cells, DC cells, and important immunosuppressive cells, myeloid-derived suppressor cells (MDSCs), were detected. More than that, the recombinant T cells in mice were functionally demonstrated to secrete the antitumor cytokine IFNγ and mediate CTL (cytotoxic T lymphocyte) responses after exposure to the human A549 lung cancer cell line. Furthermore, treatment of this Hu-PDX model with a PD-1 checkpoint inhibitor resulted in an increase in the number of CTLs, a decrease in the number of MDSCs, and inhibition of tumor growth (Meraz et al., 2019).

This kind of Hu-PDX model has made its debut in the study of tumor immunity in lung cancer. Studies have shown that after the use of immune checkpoint blockade therapy, although depleted T cells temporarily restore their function, they cannot fully recover into cytotoxic T lymphocytes, which is a pain point in the clinical use of immunosuppressants. A possible mechanism is that during the formation of exhausted T cells, their epigenetics is fixed and cell fate is locked (Pauken et al., 2016). Sanmamed et al. found a burnt-out CD8^+^ TIL subset (Ebo) specifically present in the TME in surgically resected NSCLC specimens. After that, in the Hu-PDX model, it was verified that the amplification of Ebo was dependent on the PD-1/B7-H1 pathway and was associated with the resistance of NSCLC patients to immune checkpoint inhibitors, which might serve as a potential histological biomarker (Sanmamed et al., 2021).

Another way to enable the patient-derived model to gain the ability to study tumor immunity and TME is organoid modeling. The researchers worked to achieve the co-culture of primitive lung adenocarcinoma PDO with tumor-infiltrating lymphocytes (TILs), and also verified their function with the cytotoxicity of TILs and their response to immune checkpoint inhibitor therapy (Neal et al., 2018).

### 2.6 Reprogramming of cellular metabolism

More and more studies suggest that tumorigenesis and tumor development are associated with metabolic reprogramming. The most well-known previous example is the Warburg effect. Cancer cells complete most of their energy metabolism through glycolysis to achieve high-speed energy metabolism, and it is important to generate intermediates and microenvironments that are conducive to tumor cell proliferation (Hanahan and Weinberg, 2011). Recently, researchers induced drug resistance through cisplatin exposure and found that reactive oxygen species (ROS)-mediated increase in PGC-1a might be one of the possible mechanisms of cisplatin resistance in NSCLC. PGC-1α interference or OXPHOS inhibition using metformin, which is commonly used in clinics, might delay or counteract cisplatin resistance (Cruz-Bermúdez et al., 2019).

In the field of targeted therapy, there were findings consistent with those in chemotherapy. In lung cancer cell lines and PDX models, aldo-keto reductase family 1 member B1 (AKR1B1) promoted glutathione *de novo* synthesis by activating signal transducer and activator of transcription 3 (STAT3), leading to ROS scavenging and ultimately to EGFR-TKI drug resistance. The application of a selective inhibitor of AKR1B1 in the PDX model, such as the commonly used antidiabetic drug epalrestat, also delayed the occurrence of EGFR-TKI resistance (Zhang et al., 2021b).

Another research hotspot in metabolic reprogramming is pyrimidine metabolism. Dihydroorotate dehydrogenase (DHODH) is the catalytic enzyme of the fourth step in the *de novo* pyrimidine synthesis pathway (Madak et al., 2019). The researchers firstly screened across pan-cancer genetically engineered mouse models (GEMM) and found that *in vitro* inhibition of DHODH can inhibit the growth of SCLC cell lines, and then validated the consistent antitumor effect in PDX *in vivo* experiments (Li et al., 2019). Several DHODH inhibitors are currently in preclinical and clinical studies.

Traditional molecular subtypes are often divided by driver mutations. However, adding metabolic signatures as a new dimension helps to comprehensively distinguish subtypes with therapeutically exploited characteristics. For example, for SCLC driven by different MYC family genes, further subclasses were divided according to their metabolic characteristics. It was found that for MYC-driven SCLC, adding pegylated arginine deiminase (ADI-PEG 20) to intervene arginine synthesis exhibited antitumor properties beyond conventional chemotherapy in GEMM, patient-derived cell line xenografts and PDX models (Chalishazar et al., 2019).

### 2.7 Unlocking phenotypic plasticity

It has been found that phenotypic plasticity in the tumor cell lineage is pervasive (Hanahan, 2022). There have always been dynamic and heterogeneous transitions in the process of lung cancer occurrence, development, and treatment response. Instead of intuitive linear progression and differentiation (lineage erosion), highly plastic cell state (HPCS) subpopulations can be isolated in the PDX model of lung adenocarcinoma. HPCS have characteristics of tissue stem cells and cancer stem cells (CSCs). For example, activation of the EMT program was found in the HPCS cluster, suggesting that this subgroup may be a prerequisite for EMT. Meanwhile, the plasticity of HPCS may also be closely related to the conversion from adenocarcinoma to neuroendocrine lineage. These all indicate that HPCS is closely associated with poor prognosis, resistance to targeting and chemotherapy in lung adenocarcinoma (Marjanovic et al., 2020).

### 2.8 Genome instability and mutations and non-mutational epigenetic reprogramming

Mysteries that cannot be found in other omics such as genomics may be buried in non-mutational epigenetic reprogramming. It is known transdifferentiation of lung adenocarcinoma is one of the mechanisms of resistance to targeted therapy, but because it is difficult to collect pre-transdifferentiation and post-transdifferentiation specimens for molecular biological comparison, little is known about the specific mechanism by which lung adenocarcinoma differentiates into SCLC or lung squamous cell carcinoma. Quintanal-Villalonga et al. obtained and compared the precious transdifferentiated specimens at the genomic, epigenomic, transcriptomic and proteomic levels, and found that either the neuroendocrine transformation or the squamous cell carcinoma transformation, the transformation is mainly mediated by transcriptional reprogramming, rather than gene mutation. Up-regulation of PRC2, MYC, and PI3K/AKT pathway gene expression was observed in both transdifferentiation forms. Patient-derived models played a key role in the clinical translation of this important scientific discovery: In the PDX model of EGFR-mutated lung adenocarcinoma, the combination of PI3K/AKT inhibitors and osimertinib might delay or reverse the NE transformation (Quintanal-Villalonga et al., 2021a), while EZH1/2 combined with osimertinib may resensitize the resistant squamous-like tumor to osimertinib (Quintanal-Villalonga et al., 2021b).

Even if epigenetic reprogramming does not play a direct role, it must be indispensable for its role in tumorigenesis and development. The variant NSD3 (T1232A) of the methyltransferase NSD3 caused the expression of nearby oncogenes, such as FGFR1, to be turned on by dimethylation of histone H3 lysine 36 (H3K36), which in turn drives the progression of lung squamous cell carcinoma. Depletion of NSD3 exhibited tumor growth inhibition in a PDX model of lung squamous cell carcinoma with NSD3-related variants and amplification. The researchers also obtained evidence that this PDX was sensitive to bromodomain inhibitors, suggesting the direction of further research into future therapies (Yuan et al., 2021). Similarly, the double methylation catalysis of eEF1A (eukaryotic elongation factor 1A) by METTL13 (methyltransferase-like 13) also promotes RAS-driven tumorigenesis. In the PDX model, METTL13 deletion and eEF1AK55me2 deletion benefited the growth of primary pancreatic and lung cancer tumors, while increasing tumor sensitivity to targeted drugs (Liu et al., 2019).

As another aspect of epigenomics, the role of non-coding RNAs in lung cancer cannot be ignored, and it is also reflected in patient-derived lung cancer models. Huang et al. found that circXPO1 encoded by XPO1 could serve as a potential prognostic marker and therapeutic target. High circXPO1 expression was associated with poor overall survival, and intratumoral injection of cholesterol-binding siRNA specifically targeting circXPO1 can effectively inhibit tumor growth in the PDX model (Huang et al., 2020).

Resistance to standard chemotherapy was found to be associated with an EZH2-mediated histone modification H3K27me3 in the SCLC PDX model, and EZH2 inhibitors prevent SCLC from acquiring resistance to standard chemotherapy (Gardner et al., 2017).

## 3 Technology foresight

With the wide application of targeted drugs and immune checkpoint inhibitors, the treatment of lung cancer has entered a new era of precision medicine. These emerging drugs have shown superior efficacy than conventional chemotherapy. New therapeutic paradigms, including combination therapy with immune checkpoint inhibitors, neoadjuvant, and adjuvant therapy, have led to new demands on patient-derived lung cancer models. Owing to their varied mechanisms of action, patient-derived models and protocol based on conventional chemotherapy cannot efficiently meet the clinical needs. For instance, the acquisition of immune escape during tumor growth is one of the most important hallmarks of cancer. Based on this hallmark, strategies to activate the immune system can provide significant clinical benefits to cancer patients. However, immune checkpoint inhibitors cannot directly kill tumor cells. Therefore, the advent of immunotherapy has also revolutionized the landscape of patient-derived models.

With the common aim to bring more benefit to lung cancer patients, different patient-derived models are strengthened by combining with newly developed technologies. It is possible to conduct a thorough exploration in the Frontier area of lung cancer research, and it should be noted that the different patient-derived lung cancer models are not competitors, but with a relationship of cooperation, complementarity, and even mutual transformation.

### 3.1 Allowance of gene editing to elucidate the mechanism of tumorigenesis

It is not ethical and nearly impossible to directly observe the clonal evolutionary process of lung cancer in the absence of any intervention in human tumors. Although tumor-derived cell lines could preserve the genomic and phenotypic characteristics of the primary tumors, it is a static event rather than a dynamic process, which is more in line with the situation in clinical patients. For example, tobacco exposure has been confirmed to have a close association with the development and progression of lung squamous carcinoma (The Cancer Genome Atlas Research Network, 2012), and carcinogen-induced lung squamous carcinoma mouse models have been applied in preclinical research (Yamano et al., 2016; Dwyer-Nield et al., 2021). In contrast, lung cancer in never smokers arises through the accumulation of mutations in the natural process (Zhang et al., 2021c). GEMMs that express oncogenic driver mutations such as EGFR (Politi et al., 2006), EML4-ALK (Soda et al., 2008) could generate autochthonous tumors and shed light on related targeting drug discovery. However, GEMMs require considerable time to develop, especially when multiple gene mutations are needed. The emergence of CRISPR/Cas9 technology has greatly improved efficiency (Chen et al., 2015). The PDO combined with CRISPR/Cas9 system helped to analyze the oncogenes in tumor evolution and to mimic the multi-hit oncogenesis model (Bian et al., 2018; Takeda et al., 2019).

### 3.2 Modeling of the TME

The TME has been proven to play an active role in the initiation and progression of primary *de novo* lung carcinoma and attracted much attention in recent years (Altorki et al., 2019). The lack of surrounding cellular components and cell-cell interactions within a 3D environment limit the translational potential of cell line studies (Gazdar et al., 2010). PDX and PDO models, unlike cell lines, are able to retain TME to some extent.

Conventional PDX models are generated in immunodeficient mice, resulting in the inability to explore the functions of immune cells in the TME and the possible mechanisms of immunotherapy. The aforementioned Hu-PDX is an important attempt to solve this problem. Besides that, TME including vasculature, fibroblasts, and the extracellular matrix are also currently lacking in conventional organoid cultures. This has been partly resolved by the establishment of co-cultures with cancer-associated fibroblasts (CAFs) (Fiorini et al., 2020). Researchers isolated and extracted patient-derived fibroblast (PDF) from lung cancer biopsy samples for culture expansion and established a PDF library. By coculturing tumor cell lines with PDF and testing the sensitivity of targeted therapy drugs, it was found that PDF affected the efficacy of EGFR and ALK inhibitors through different degrees of activation of HGF-MET and FGF-FGFR signaling pathways. Based on this, CAFs were divided into three subtypes. This is a patient-derived CAF biobank focused on the heterogeneity of CAF and TME (Hu et al., 2021a).

### 3.3 3D cell culture technologies

Strategies including submerged Matrigel culture, microfluidic 3D culture, and air-liquid interface culture have been developed to probe the interactions between tumor cells and immune cells in the PDO model (Yuki et al., 2020). Matrigel culture media provides natural extracellular matrix (ECM)-based hydrogels with 3D inter-connected nanofiber networks for *in vitro* 3D cell culture leading to organoid and spheroid formation. The self-assembly nanofibers structures of the fibronectin and collagen proteins from ECM together with a large amount of trapped water molecules facilitate the suitable mechanical properties and network structures for 3D cell culture. It has been reported that the growth kinetics, differentiation, and drug response of the PDOs could possibly be modulated by the stiffness of extracellular matrix such as the Matrigel concentration (Li and Izpisua Belmonte, 2019).

Compared with animal models, PDO is the simple mimicking system lacking stroma, blood cells, and immune cells. With the development of the 3D cell culture technologies, co-culture of tumor cells and blood cells or even immune cells are feasible. Researchers have reported that the co-culture of autologous peripheral blood lymphocytes with tumor organoids could induce the proliferation of tumor-reactive T cells (Dijkstra et al., 2018). Autologous tumor organoids would be recognized and killed by reactive T-cells, whereas the autologous healthy organoids not, indicating the ability of this platform for assessing the efficiency of T-cell mediated tumor killing (Cattaneo et al., 2020). Tumor organoids are gradually used in assessing the effectiveness as well as adverse reactions of immunotherapeutic strategies (Schnalzger et al., 2019).

Moreover, the cutting-edge organ-on-a-chip technology combining 3D culture systems and microfluidic technology has also emerged. Microfluidics allows spatiotemporal controls over fluids within the micrometer-sized channels, which could modulate the cell culture under controlled conditions or signaling gradients, and also could simulate the shear force of the fluid on the cell growth and polarization. Construction on the micron scale allows for a high degree of preservation of organ structure and microenvironment (Huang et al., 2021; Liu et al., 2022). Precise spatiotemporal controls and continuous monitoring are achieved by combining with artificial intelligence and innovative imaging technologies such as mico-computed tomography (Zheng et al., 2016). Advanced 3D cell culture technologies could facilitate the intricate modulation of the PDO models, which could improve the PDO system more mimicking the true tumor environment.

### 3.4 Biobank and high-throughput drug screening

Even with the advance in innovative technologies and understanding of the molecular mechanisms of tumors, the failure rate of phase I-III trials remains extremely high (Wong et al., 2018). These pre-clinical models are pivotal to bridging the translational gap between drug development and clinical application and could help to improve the translation from bench to bedside. The significant advantages of traditional cell lines are low cost, simple and rapid culture process. The established large-scale public resource databases will benefit researchers around the world, such as the famous The Cancer Cell Line Encyclopedia (CCLE) (Barretina et al., 2012), and the Genomics of Drug Sensitivity in Cancer (GDSC) (Yang et al., 2013). Lung cancer cell lines have been widely used in high-throughput screening of potential therapeutic agents before animal experimentation is carried out (Wilding and Bodmer, 2014). However, divergent or even contradictory responses are observed among cell lines, which may be attributed to acquired genetic changes and differences in culture conditions (Zhang et al., 2015).

Patient-derived lung cancer models formed by primary tumor cells are shown to recapitulate the parental tumor characteristics, and the drug testing results are more truthful and reliable (Abdolahi et al., 2022). However, the accelerating development of new drugs and the rise of combination therapy require not only the accuracy of results but also a high-throughput solution. Gao et al. performed a high-throughput drug screening using ∼1,000 PDX models with a diverse set of driver mutations and validated the feasibility of this approach by correlating genomic information with observed efficacy (Gao et al., 2015). The concept of PDX biobank and PDX clinical trial (PCT) was proposed and this experimental paradigm showed great potential for applications in drug screening.

Similarly, an automated and high-throughput screening approach to identify drug sensitivity in PDO has been established and its efficiency, rapidity, and high fidelity demonstrated great commercial value in precision medicine and preclinical drug screening (Phan et al., 2019).

### 3.5 Approaching personalized precision medicine

With the dramatic development of multi-omics technology and translational medicine, the diagnosis and clinical treatment of lung cancer have entered the era of population-based precision medicine (Yang et al., 2022). However, the conclusions from phase III trials based on general populations are not equally applicable to all patients. Targeted therapy, as a representative example of precision medicine in lung cancer, the overall objective response rates are no more than 80% (Soria et al., 2018; Shaw et al., 2020). There is still some distance away from achieving personalized precision medicine. Patient-derived models are expected to develop individualized treatment plans and resolve the “last kilometer” problems.

In addition to the identification of initial treatment options, patient-derived models can also help in revealing the resistance mechanisms (Nie et al., 2022) and PDX models captured at the moment of maximal tumor shrinkage during exposure to antitumor agents provided a valuable opportunity for exploring the possible resistance mechanisms before real resistance occurs (Lupo et al., 2020). Single-cell sequencing on the residual PDX models has demonstrated its potential to reveal the evolutionary and selective mechanisms under the selection pressure of anti-cancer drugs (Rambow et al., 2018).

Compared to PDX models, the establishment of PDO is less time-consuming and may therefore have the potential to elucidate resistance mechanisms and inform clinical decisions (Hu et al., 2021b). With single-cell RNA sequencing of hepatobiliary tumor organoids, the collaboration of intratumoral specific subpopulations was identified as the cause of drug resistance (Zhao et al., 2021b). Overall, it is very tempting to implement organoid technology in basic and clinical research to make the idea of personalized precision medicine become a reality. However, one has to admit that the success rate of culturing organoids is far from satisfactory for clinicians. It varies across tumor types and can be affected by many factors, especially tumor cellularity and the available starting material (Yan et al., 2018; Ooft et al., 2021). A high and stable culture success rate is a necessary and important step toward the clinical application of organoids (Dijkstra et al., 2020).

## 4 Conclusion

The *Hallmarks of Cancer* summarizes the complex biological features of tumors into a constantly evolving set of underlying principles and has made a significant impact on basic and clinical tumor research. These patient-derived models are essential for bridging the translational gap between molecular mechanisms and clinical translation. However, from our perspective, there is still a large gap between the existing patient-derived models and ideal *in vitro* lung cancer models. Existing models have been formulated considering only the tumors' own biological behaviors, which makes the models too simple to mimick the true condition of the tumor. In addition, no standard protocol for patient-derived models has been established to guarantee the quality of basic and clinical research. Therefore, the establishment of unified standards in patient-derived models is paramount to advancing the field. Moreover, there are too many confounding human factors in the culture process, and future investigations should focus on shifting all of the processes towards automation, high throughput, and digitization.

Collectively, each of these models has advantages and disadvantages that must be considered. Advanced 3D cell culture technologies is promising in the modulation of the PDO models to more simulate the patients TME. While there is no ideal model that can fully recapitulate the phenotypes and genotypes of the primary patient tumor, choosing the most appropriate model for addressing scientific questions is always the top priority.

## Glossary

ADC: antibody-drug conjugates
AKR1B1: aldo-keto reductase family 1 member B1
ALK: anaplastic lymphoma kinase
bFGF: basic fibroblast growth factor
CAFs: cancer-associated fibroblasts
CCLE: Cancer Cell Line Encyclopedia
CDK: Cyclin-dependent Kinase
CSCs: cancer stem cells
CTC: circulating tumor cell
CTL: cytotoxic T lymphocyte
DDR: DNA damage response
DHODH: Dihydroorotate dehydrogenase
DSB: double strand break
eEF1A: eukaryotic elongation factor 1A
METTL13: methyltransferase-like 13
EGFR: epidermal growth factor receptor
EMT: epithelial-mesenchymal transition
GDSC: Genomics of Drug Sensitivity in Cancer
GEMM: genetically engineered mouse models
HIP1: huntingtin interacting protein 1
HPCS: highly plastic cell state
IGF-1R: insulin-like growth factor-1 receptor
ITA: itraconazole
MDSCs: myeloid-derived suppressor cells
NSCLC: non-small-cell lung carcinoma
PCT: PDX clinical trial
PDF: patient-derived fibroblast
PDO: patient-derived organoids
PDX: patient-derived tumor xenograft
ROS: reactive oxygen species
RPPA: reverse-phase protein array
SCLC: small-cell lung carcinoma
STAT3: signal transducer and activator of transcription 3
TILs: tumor-infiltrating lymphocytes
TKIs: tyrosine kinase domains
TME: tumor microenvironment
VEGF: vascular endothelial growth factor
